# Effects of Macrophage Depletion on Sleep in Mice

**DOI:** 10.1371/journal.pone.0159812

**Published:** 2016-07-21

**Authors:** Conner Ames, Erin Boland, Éva Szentirmai

**Affiliations:** 1 Elson S. Floyd College of Medicine, Department of Biomedical Sciences, Washington State University, Spokane, Washington, United States of America; 2 Sleep and Performance Research Center, Washington State University, Spokane, Washington, United States of America; University of Oxford, UNITED KINGDOM

## Abstract

The reciprocal interaction between the immune system and sleep regulation has been widely acknowledged but the cellular mechanisms that underpin this interaction are not completely understood. In the present study, we investigated the role of macrophages in sleep loss- and cold exposure-induced sleep and body temperature responses. Macrophage apoptosis was induced in mice by systemic injection of clodronate-containing liposomes (CCL). We report that CCL treatment induced an immediate and transient increase in non-rapid-eye movement sleep (NREMS) and fever accompanied by decrease in rapid-eye movement sleep, motor activity and NREMS delta power. Chronically macrophage-depleted mice had attenuated NREMS rebound after sleep deprivation compared to normal mice. Cold-induced increase in wakefulness and decrease in NREMS, rapid-eye movement sleep and body temperature were significantly enhanced in macrophage-depleted mice indicating increased cold sensitivity. These findings provide further evidence for the reciprocal interaction among the immune system, sleep and metabolism, and identify macrophages as one of the key cellular elements in this interplay.

## Introduction

Reciprocal interactions between sleep and the immune system has long been recognized. The activation of the immune system modulates sleep while sleep abnormalities affect immune function. Sickness behavior is associated with lethargy, fatigue and profound alterations in sleep-wake activity. Pathogens alter sleep, in part, through inflammatory mechanisms. Several cytokines, including the pro-inflammatory cytokines interleukin (IL) 1α, IL1β, IL6, interferon (IFN)α, IFNγ, tumor necrosis factor (TNF)α, and TNFβ, modulate sleep-wake behavior (reviewed in [1]). Sleep loss impairs immune competence. Animal studies indicate that fragmented or attenuated sleep is associated with decreased survival after microbial challenge [2]. Chronic sleep deprivation results in intestinal bacterial overgrowth, microbial penetration into lymph nodes, and lethal systemic bacterial invasion in rats [3]. In humans, chronic insufficient sleep is associated with various inflammatory pathologies including cardiovascular diseases, obesity and metabolic syndrome. These pathologies are also characterized by increased levels of endogenous pro-inflammatory cytokines such as IL1β and TNFα [4].

The role of specific cellular elements in the interactions between sleep and the immune system is not completely understood. Previous studies have implicated macrophages in the production and metabolism of somnogenic substances after bacterial [5, 6] or viral challenge [7]. Macrophages are present in nearly every tissue; tissue-resident macrophages adapt to local microenvironments and acquire tissue-specific phenotypes and functions. Macrophage activation may lead to M1 (classically activated) phenotype, M2 (alternatively activated) phenotype or intermediate phenotypes with mixed M1 and M2 characteristics (reviewed in [8]). Indirect evidence suggests that the activation of both macrophage phenotypes may signal for increased sleep. For example, M1 macrophages secrete pro-inflammatory cytokines [reviewed in 8], many of which stimulate sleep (reviewed in [1]). M2 macrophages play an integral role in the activation of brown adipose tissue (BAT) [9, 10]; activated BAT is a source of sleep-promoting signals [11].

Based on the above evidence, we hypothesized that macrophages play a role in integrating immune functions with sleep-regulatory mechanisms. We tested this hypothesis by determining the ability of macrophage-depleted mice to mount rebound sleep responses after sleep loss and to maintain sleep in cold environment. We used clodronate-containing liposomes (CCL) for macrophage depletion. Macrophages phagocytose CCL, which is subsequently degraded by lysosomal phospholipases leading to the release of clodronate. Free clodronate accumulates intracellularly and induces the apoptosis of macrophages by eliciting irreversible metabolic damage [12]. Free clodronate released from disintegrated macrophages has a short, ~15 min, half-life in circulation and does not enter other cells in its free form [13]. The biological effects of CCL treatment show two distinct phases. During the first 24 h after CCL injection, the acute effects of bioactive substances that are released from disintegrating macrophages dominate. Subsequently, a macrophage-depleted steady state develops with all the consequences of suppressed macrophage function [13].

We investigated both the immediate, acute effects of CCL treatment on sleep and thermoregulation and the integrity of sleep mechanisms during the following macrophage-depleted state. We report here that acute systemic injection of CCL induces transient increases in non-rapid-eye movement sleep (NREMS) and body temperature, with a simultaneous decrease in rapid-eye movement sleep (REMS) and electroencephalographic slow-wave activity (EEG SWA). Macrophage-depleted mice display attenuated recovery sleep response after sleep deprivation and increased sensitivity to cold challenge. These findings provide evidence for a role of macrophages in the regulation of sleep-wake activity and body temperature.

## Methods

### Animals

Adult, male C57BL/6 mice purchased from the Jackson Laboratories, Inc., were housed in individual cages within sound-attenuated, temperature-controlled isolation chambers on a 12:12 light-dark cycle (lights on at 4 am) at 22°C ambient temperature (except where stated otherwise). All animal work was conducted in compliance with the recommendations in the Guide for the Care and Use of Laboratory Animals of the National Institutes of Health. All animal protocols were approved by the Institutional Animal Care and Use Committees at Washington State University, and all efforts were made to minimize suffering.

### Surgery

Using ketamine-xylazine anesthesia (87 and 13 mg/kg, respectively), 3–4 months old mice were implanted with cortical EEG electrodes, placed over the frontal and parietal cortices, and electromyographic (EMG) electrodes in the dorsal neck muscles. The EEG and EMG electrodes were connected to a pedestal, which was attached to the skull with dental cement. Temperature-sensitive transmitters were implanted intraperitoneally (i.p.) for telemetry recordings of body temperature and motor activity. Mice were allowed to recover from surgery for 7–10 days before baseline recordings started. Food and water were available *ad libitum* throughout the experiments. The animals were fed with regular lab chow (Harlan Teklad, Product No. 2016), in which fat, proteins, and carbohydrates provided 12%, 22%, and 66% of calories, respectively.

### Sleep-wake recordings and analyses

EEG and EMG signals were digitized at 256 Hz collected by a Grass Model 15 Neurodata amplifier system (Grass Instrument Division of Astro-Med, Inc., West Warwick, RI) using SleepWave software (Biosoft Studio, Hersey, PA). Sleep-wake states were scored visually off-line in 10-s epochs by defining the vigilance states as NREMS, REMS and wakefulness (W) according to standard criteria as described previously [14]. Time spent in W, NREMS and REMS was tabulated in 2-, 12- and 24-h blocks. On-line fast Fourier transformation was performed on EEG power data from each artifact-free 10-s segment. EEG power data in the range of 0.5 to 4.0 Hz during NREMS were used to compute EEG SWA. EEG SWA data were averaged across the entire 24-h recording period on the baseline day for each mouse to obtain a reference value. EEG SWA values for baseline and experimental days were expressed as a percentage of this reference value (units). The results were further averaged in 2 and 24-h bins. In experiment 2, EEG SWA data of one control animal was excluded from statistical analysis due to numerous EEG artifacts.

### Telemetry recordings

Core body temperature and locomotor activity were recorded by MiniMitter telemetry system (Starr Life Sciences Corp., Oakmont, PA) using VitalView software. Temperature and activity values were collected every 1 and 10 min, respectively, and were averaged over 2-, 12- or 24-h time blocks. In experiment 2, data from two CCL-treated mice had to be excluded from the analysis due to transmitter malfunction.

### Experimental procedures

#### Experiment 1: Acute effects of CCL treatment on sleep, body temperature and motor activity

On the baseline day (day 1), all mice were injected i.p. with saline 5–10 min before the beginning of the dark phase. Sleep-wake activity, motor activity and body temperature were recorded for 24 h after the treatment. On the next day (day 2) 5–10 min before the beginning of the dark period, the experimental group of mice (n = 14) was injected with CCL i.p. (1.2 mg/mouse in 0.2 ml volume, FormuMax Scientific, Inc, Palo Alto, CA); the control group (n = 10) received saline. Recordings started immediately after the injections for 24 h. To test if CCL treatment caused any long-term changes in sleep, all mice were injected with saline on day 8, i.e., six days after the injection of CCL, and recordings were repeated for 24 h. Differences in sleep, activity and temperature were calculated between day 2 and day 1 as well as between day 8 and day 1.

#### Experiment 2: Effects of sleep deprivation on sleep, activity and body temperature in macrophage-depleted mice

Ten control and eight CCL-pretreated mice were used from the previous experiment. Seven days after the CCL and saline injections baseline sleep, locomotor activity and body temperature were recorded for 24 h starting from dark onset. On the next day, mice were sleep-deprived by gentle handling during the last 6 h of the light period. Sleep, body temperature and motor activity recordings were started immediately after the end of the sleep deprivation at the beginning of the dark period (recovery day). For both groups, differences in sleep, activity and temperature were calculated between the recovery and baseline day.

#### Experiment 3: Effects of 10°C cold exposure in macrophage-depleted mice

In mice, intact thermogenic activity of BAT is required for maintaining body temperature (reviewed in [15]) and sleep [11] in cold environment. Since macrophage depletion impairs the thermogenic activity of BAT [9], we hypothesized that intact macrophage function is also required for maintaining sleep in the cold. New groups of CCL- (n = 5) and saline-pretreated animals (n = 7) were subjected to cold exposure test 1 week after the pretreatments. Sleep, body temperature and activity were recorded on the baseline day in the home cages at 22°C for 24 h then the thermostat was set to 10°C 5 min before the beginning of the dark period for an additional 24-h period. Ambient temperature reached 10°C within 30 min after setting the thermostat.

### Immunohistochemistry

To verify macrophage depletion after the CCL treatment, we performed immunohistochemical staining of the visceral white adipose tissue and liver by using an antibody against F4/80 protein, a specific marker for macrophages. Animals were sacrificed using Isoflurane overdose and transcardially perfused with PBS followed by 4% formaldehyde. Formalin-fixed paraffin-embedded visceral adipose tissues were sectioned (5 μm thick), deparaffinized in xylene, and rehydrated prior to antigen unmasking in proteinase K buffer (Sigma-Aldrich). Sections were blocked with 3% hydrogen peroxide and 5% goat serum and then incubated with rat anti-mouse F4/80 primary antibody (1:200; AbD Serotec, Raleigh, NC) overnight. Sections were then incubated with biotinylated goat anti-rat secondary antibody (1:500; Jackson ImmunoResearch Laboratories, Inc., West Grove, PA) for 2 hours followed by incubation in Avidin-Biotin Complex and developed using 3,3’-diaminobenzidine as substrate (Vector Laboratories, Burlingame, CA). All incubations took place in a humidity chamber at room temperature. Liver sections were counterstained with cresyl violet. Sections were examined using a Leica DM 2000 LED light microscope and images were captured using a Leica ICC50 W Microscope Camera and Leica Imaging Software. F4/80 positive cells were counted on 10 high power (20x) fields per slide (n = 3/condition).

### Statistics

NREMS, REMS and W amounts, EEG SWA, average body temperature, motor activity were calculated in 2-, 12 and 24-h blocks. Two-way mixed analysis of variance (ANOVA) was performed across 24 h (independent measure: treatment, repeated measure: time). When ANOVA indicated significant effects, Student-Newman-Keuls test was used as the *post hoc* test for all experiments. NREMS, REMS and W episode numbers and average episode durations were calculated for the 12-h dark and 12-h light periods and comparisons were made between experimental groups by performing Student’s t-tests. An α-level of *P* < 0.05 was considered to be significant. Detailed statistical results are in Tables 1–3.

**Table 1:** Statistical results: The effects of clodronate-containing liposome administration on wakefulness, non-rapid-eye movement sleep (NREMS), rapid-eye movement sleep (REMS), slow-wave activity (SWA) of the electroencephalogram, body temperature and motor activity during the 24-h after injection.

	Treatment	Time	Treatment x Time
**Wake (min)**	F(1,13) = 14.5, p < 0.05	F(11,143) = 31.3, p < 0.05	F(11, 143) = 12.6, p < 0.05
**NREMS (min)**	F(1, 13) = 31.5, p < 0.05	F(11,143) = 23.6, p < 0.05	F(11, 143) = 14.0, p < 0.05
**REMS (min)**	F(1, 13) = 160.6, p < 0.05	F(11,143) = 43.7, p < 0.05	F(11, 143) = 5.9, p < 0.05
**SWA (Units)**	F(1, 13) = 26.4, p < 0.05	F(11,143) = 11.3, p < 0.05	F(11, 143) = 10.2, p < 0.05
**Body Temperature (°C)**	F(1, 13) = 24.0, p < 0.05	F(11,143) = 29.2, p < 0.05	F(11, 143) = 11.8, p < 0.05
**Activity (Count)**	F(1, 13) = 219.2, p < 0.05	F(11,143) = 13.8, p < 0.05	F(11, 143) = 14.5, p < 0.05

**Table 2:** Statistical results: sleep, body temperature and activity responses of control mice after sleep deprivation.

	Treatment	Time	Treatment x Time
**Wake (min)**	F(1,8) = 55.3, p < 0.05	F(11,88) = 31.9, p < 0.05	F(11,88) = 5.9, p < 0.05
**NREMS (min)**	F(1,8) = 40.7, p < 0.05	F(11,88) = 28.8, p < 0.05	F(11,88) = 6.1, p < 0.05
**REMS (min)**	F(1,8) = 24.3, p < 0.05	F(11,88) = 15.8, p < 0.05	F(11,88) = 3.7, p < 0.05
**SWA (Units)**	F(1,7) = 2.5, n.s.	F(11,77) = 16.0, p < 0.05	F(11,77) = 24.3, p < 0.05
**Body Temperature (°C)**	F(1,8) = 1.5, p < 0.05	F(11,88) = 23.9, p < 0.05	F(11,88) = 1.2, p < 0.05
**Activity (Count)**	F(1,8) = 16.1, p < 0.05	F(11,88) = 12.2, p < 0.05	F(11,88) = 3.2, p < 0.05

**Table 3:** Statistical results: sleep, body temperature and activity responses of macrophage-depleted mice after sleep deprivation.

	Treatment	Time	Treatment x Time
**Wake (min)**	F(1,6) = 0.27, n.s.	F(11,66) = 13.0, p < 0.05	F(11,66) = 1.2, n.s.
**NREMS (min)**	F(1,6) = 0.0, n.s.	F(11,66) = 11.8, p < 0.05	F(11,66) = 0.7, n.s.
**REMS (min)**	F(1,6) = 7.5, p < 0.05	F(11,66) = 10.3, p < 0.05	F(11,66) = 3.9, p < 0.05
**SWA (Units)**	F(1,6) = 3.6, n.s.	F(11,66) = 15.2, p < 0.05	F(11,66) = 11.2, p < 0.05
**Body Temperature (°C)**	F(1,4) = 140.2, p < 0.05	F(11,44) = 23.2, p < 0.05	F(11,44) = 0.6, n.s.
**Activity (Count)**	F(1,4) = 0.7, n.s.	F(11,44) = 7.5, p < 0.05	F(11,44) = 1.3, n.s.

## Results

### Verification of macrophage depletion

**Fig 1:**
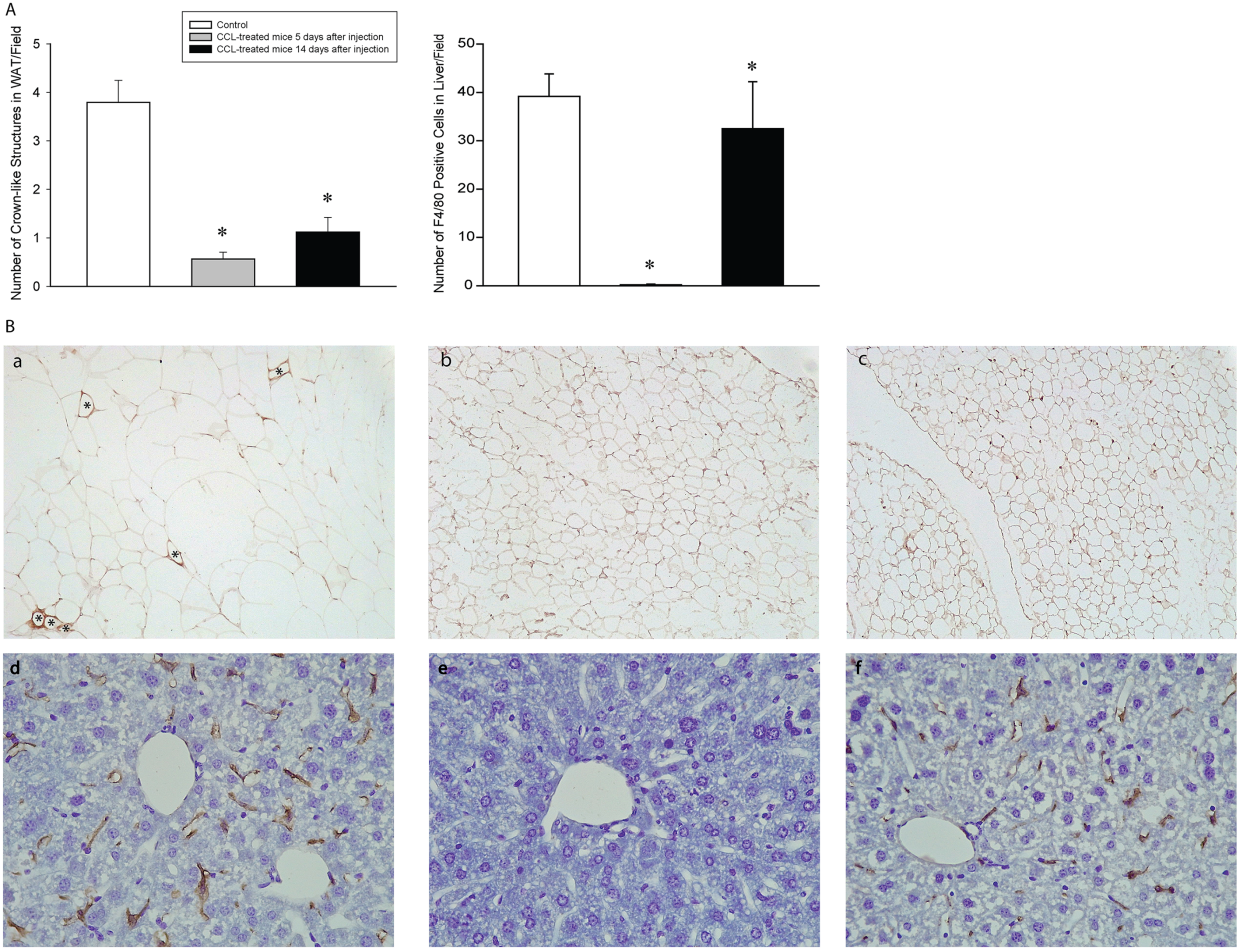
Intraperitoneal injection of clodronate-containing liposomes depletes macrophages in visceral white adipose tissue and liver in mice. A: the total number of crown-like structures/10 visual fields and the number of F4/80 positive cells/field in liver. Asterisks denote significant differences from control. B: Representative images of visceral white adipose tissues from control (a) and clodronate-containing liposome-treated mice 5 (b) and 14 days (c) after injections. F4/80 positive macrophages around adipocytes (crown-like structures) are indicated by asterisks. Representative images of liver from control (d) and clodronate-containing liposome-treated mice 5 (e) and 14 days (f) after injections.

Crown-like structures in the adipose tissue are composed of aggregate of macrophages around individual adipocytes [16]. Macrophage depletion by using CCL is known to reduce the number of crown-like structures in white adipose tissue as well as Kupffer cells in liver [17, 18]. We found that mice treated with CCL had significantly decreased number of crown-like structures in their visceral adipose tissue 5 and 14 days after CCL administration. In the liver, Kupffer cells were completely eliminated by day 5 after CCL treatment. By day 14, Kupffer cell number recovered to a degree but remained still significantly below control levels (Fig 1). These findings verify that systemic administration of CCL significantly suppressed the number of at least two main peripheral F4/80-positive macrophage populations.

### Acute effects of CCL on sleep, body temperature and motor activity

**Fig 2:**
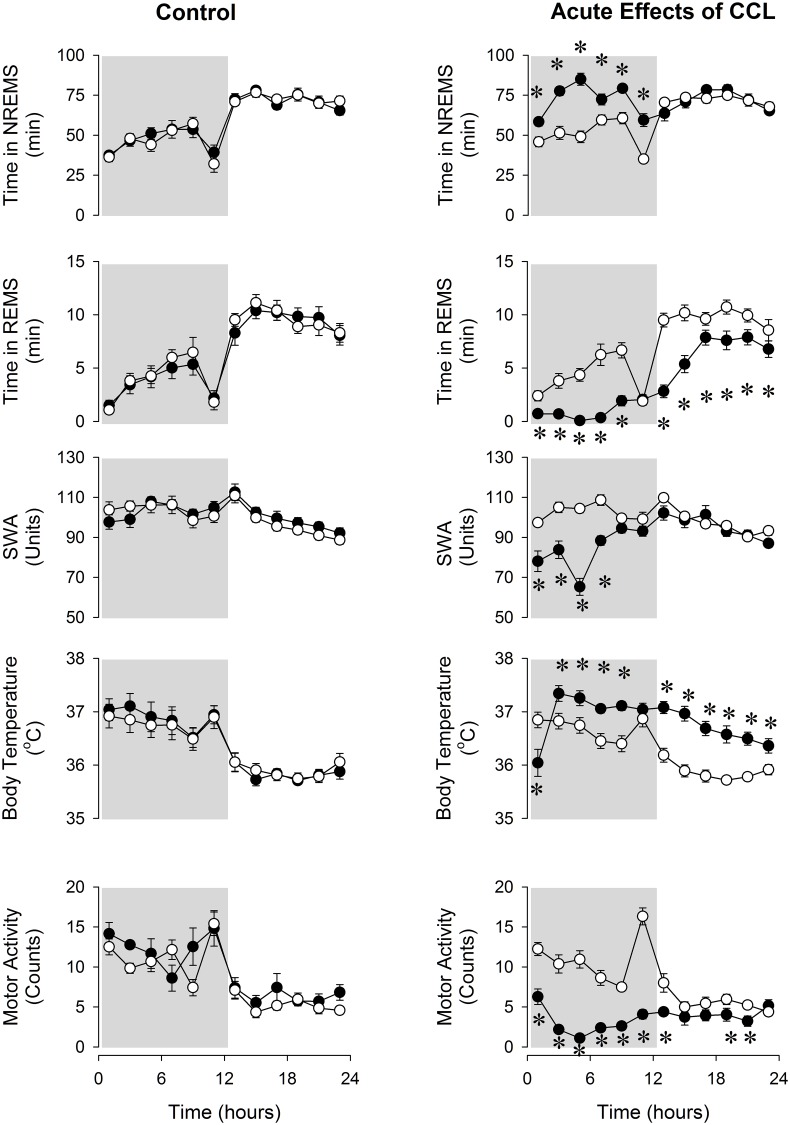
Non-rapid-eye-movement sleep (NREMS), rapid-eye-movement sleep (REMS), electroencephalogram slow-wave activity (SWA), body temperature and motor activity in control and clodronate-containing liposome (CCL)-treated mice. Open symbols: baseline day (saline injection for both groups), solid symbols: experimental day (CCL injections for the CCL-treated group and saline injection for the control group). Data are binned in 2-h time blocks. Time “0”: time of the injections at ZT12. Dark shaded areas: dark period. Error bars: standard error. Asterisks denote significant differences between baseline and treatment days (*post hoc* Student-Newman-Keuls test).

In the control group, injection of saline had no effect on sleep-wake activity, body temperature or motor activity (Fig 2). Intraperitoneal injection of CCL caused acute increases in NREMS and suppression of REMS, SWA and motor activity in the experimental group. NREMS increased by ~43% during the dark period (Fig 2, Table 1). This was due to increased number of NREMS episodes after CCL treatment (63.7 ± 3.4 vs 81.9 ± 4.6 episodes per 12 h, baseline vs. CCL; p < 0.05); the average duration of NREMS episodes was not affected significantly. REMS was suppressed across the entire 24-h period by ~47% following the injection of CCL due to the decreased number of REMS episodes (56.9 ± 2.4 vs 31.4 ± 1.6, per 24 h, baseline vs. CCL; p < 0.05).

EEG SWA was decreased for 8 h after CCL treatment and motor activity was suppressed for the entire 24-h recording period. Body temperature showed biphasic changes. An initial drop of ~ 0.7–1°C in the first two hours was followed by a prolonged hyperthermic phase lasting to the end of the recording period (Fig 2, Table 1).

By day 6 after CCL injection, NREMS, REMS, motor activity and body temperature returned to baseline, while SWA remained significantly suppressed (NREMS: 780 ± 27 vs. 813 ± 35 min/24 h, REMS: 88 ± 5 vs. 80 ± 8 min/24 h, baseline vs. day 6).

### Effects of sleep deprivation on sleep and body temperature in macrophage-depleted mice

**Fig 3:**
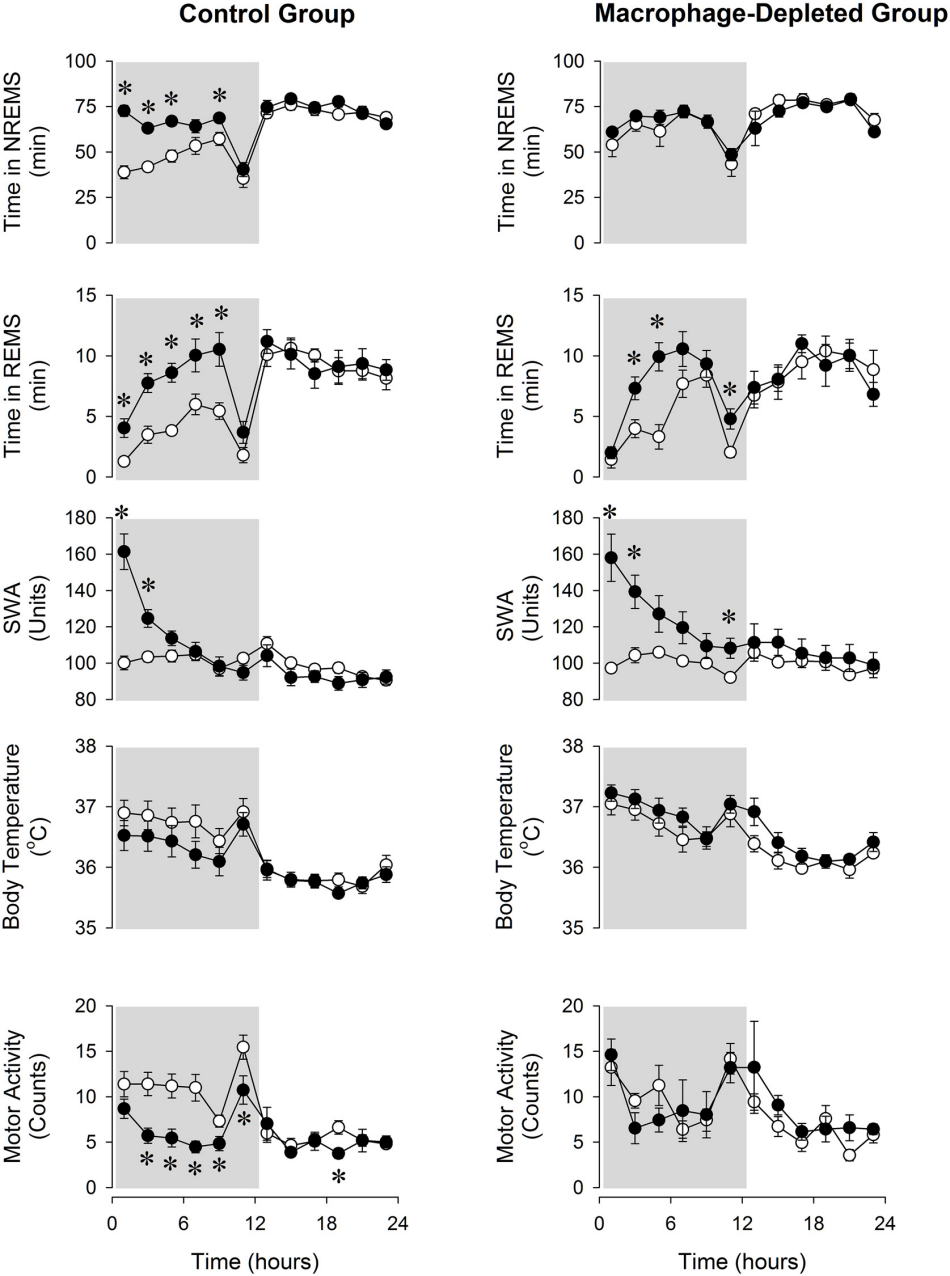
NREMS, REMS, EEG SWA, and body temperature in control and macrophage-depleted mice on the baseline day (open symbols) and after 6 h sleep deprivation (solid symbols). See legends to Fig 2 for details.

Baseline sleep-wake activity showed a slight but significant difference between the control and macrophage-depleted mice, with more NREMS in macrophage-depleted animals [F(1,16) = 7.3, p<0.05]. Sleep deprivation induced significant rebound increases in NREMS and REMS and slight decreases in body temperature in control mice in the first 12 h on the recovery day (Fig 3, Table 2). These measures returned to normal during the second 12-h period. EEG SWA increased significantly in the first 4 h after sleep deprivation followed by a decrease during the subsequent light period. Changes in motor activity mirrored the sleep effects; during the first 12 h activity was significantly suppressed.

Rebound sleep responses to sleep deprivation were significantly attenuated in macrophage-depleted mice (Fig 3, Table 3). The rebound NREMS increase observed in controls was completely abolished in macrophage-depleted mice. There was no significant difference in baseline body temperature between control and macrophage-depleted mice [F(1,14) = 0.4, p>0.05]. There was no significant difference in the REMS, EEG SWA and body temperature responses to sleep deprivation of the two groups.

### Effects of 10°C cold exposure in macrophage-depleted mice

**Fig 4:**
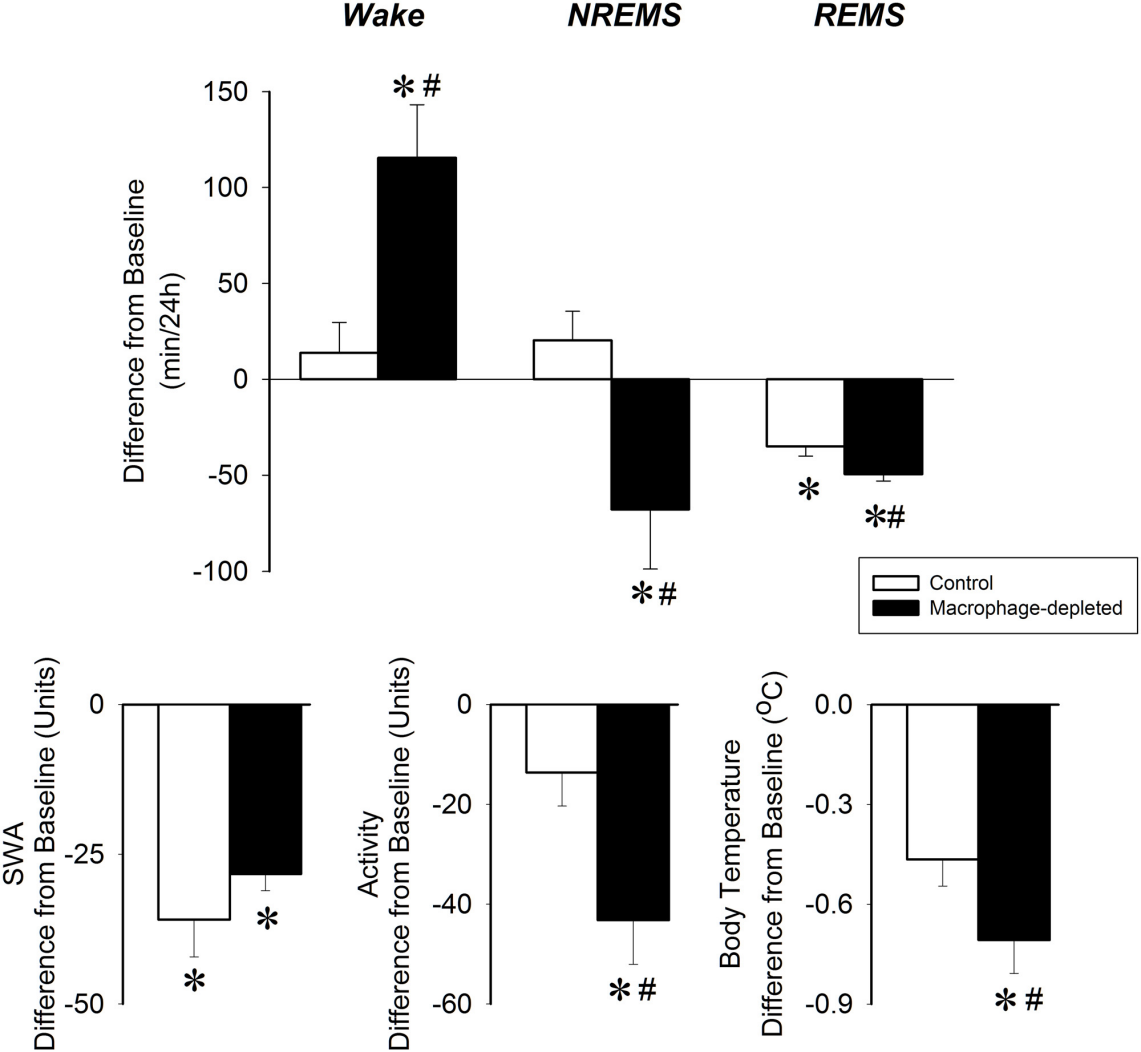
Changes in wakefulness, NREMS, REMS, EEG SWA, motor activity and body temperature in control (white bars) and CCL-treated (black bars) mice during 24 h of 10°C cold exposure. Data are shown in 24-h time blocks. Asterisks denote significant differences from baseline, # denote significant difference between control and CCL-treated groups.

There was no significant difference in the amount NREMS or REMS on the pre-cold exposure baselines between the control and macrophage-depleted mice [F(1,10) = 3.5, p>0.05 for NREMS, F(1,11) = 0.09, p>0.05 for REMS]. Lowering the ambient temperature from 22°C to 10°C significantly decreased REMS in control mice, while the amount of wakefulness and NREMS remained unchanged during the 24-h recording period (Fig 4). There was a tendency towards decreased body temperature and motor activity.

In macrophage-depleted mice, the effects of cold exposure on sleep-wake activity and body temperature were more robust as compared to controls. In addition to decreases in REMS, NREMS was also suppressed and wakefulness increased. Both motor activity and body temperature were significantly reduced in macrophage-depleted mice. EEG SWA was reduced in both groups of mice without significant difference between the groups.

## Discussion

Interactions among sleep, metabolism and the immune system have been described in health and disease. The role that various tissues, mechanisms and cellular elements mediating this interplay, however, remains unclear. Our present findings indicate that intact macrophage function is required for homeostatic sleep increases after sleep loss and for maintaining normal sleep in cold environment. Macrophages have already been implicated in sleep responses to microbial challenges [5, 6, 7]. For example, suppression of pro-inflammatory cytokine production/release from macrophages by genetic or pharmacological tools attenuates influenza virus-induced sleep enhancement in mice [7]. It had not been studied, however, if intact macrophage function contributes to sleep signaling under non-inflammatory conditions. In the present study, we demonstrated that fundamental sleep-promoting mechanisms are impaired in macrophage-depleted mice.

Sleep deprivation elicited rebound increases in NREMS, REMS and EEG SWA in control mice. These responses are similar to those previously reported for C57BL/6 mice [19, 20, 21]. Macrophage-depleted mice failed to mount rebound NREMS increases after sleep deprivation indicating the insufficiency of homeostatic sleep regulation. There was a slight but significant difference in the baseline amounts of wake and NREMS between the control and macrophage-depleted mice which could account, at least in part, for the attenuated NREMS response. It is unlikely, however, that the almost complete lack of rebound NREMS increases in the macrophage-depleted animals is due to a ceiling effect. In previous studies, we observed a significant increase in NREMS after sleep-inducing stimuli in mice with similarly high baseline NREMS amounts during the dark phase [22]. REMS rebound, while slightly attenuated, was not significantly affected in macrophage-depleted animals. EEG SWA increases after sleep loss, which is often regarded as one of the measures of sleep pressure, was also preserved. These indicate that the mechanisms which are responsible for the compensatory increases in NREMS time are different from those that underpin homeostatic SWA and REMS responses. This supports the notion that the amount of NREMS and EEG SWA are independently regulated (reviewed in [23]). Our results indicate that signals from macrophages contribute to the generation and maintenance of NREMS.

Environmental factors greatly affect sleep. An important aspect of sleep regulation is to bring about adaptive changes in sleep in response environmental influences and maintain sleep amounts optimal for the given environmental conditions. Our findings in control mice are in line with prior observations that REMS is decreased in mice and rats at subthermoneutral ambient temperatures [24–28]. During REMS, thermoregulatory reflexes such as the regulated heat production are inhibited and the animals become poikilothermic (reviewed in [29]). Decreases in REMS are, therefore, adaptive when mice are exposed to cold. Macrophage-depleted mice showed an augmented suppression of REMS, greatly reduced NREMS and increased wakefulness as well as a significant drop in body temperature. Increased wakefulness and declining body temperature are indicative of increased cold-sensitivity. Our results indicate that macrophage function is required for normal cold-sensitivity and maintaining optimal sleep at low ambient temperatures.

Administration of CCL is commonly used to deplete macrophages in vivo (e.g., [9, 17, 30–32]). 24–48 h after i.p. CCL treatment, a steady state of physical depletion of macrophages and monocytes occurs in the bone marrow, spleen, liver, lymph nodes and peritoneal cavity lasting for 10–14 days [33]. During this period, the number of functional macrophages is reduced to ~30% of normal [33]. We also show that 5 days after CCL treatment, F4/80-positive macrophages were completely depleted from the liver and suppressed to less than 15% of control in the white adipose tissue. This verifies that our delivery of CCL was adequate. Macrophage numbers did not return to normal on day 14. Our sleep deprivation and cold exposure experiments were carried out during the macrophage-depleted state, 7 days after CCL treatment.

CCL depletes both M1 and M2 macrophage pools [30, 34, 35] thus the relative contribution of the two main macrophage populations to sleep cannot be determined from our study. Extensive evidence suggests, however, that sleep-inducing signals may arise from both M1 and M2 macrophages. M1 macrophages are the main source of circulating pro-inflammatory cytokines during inflammation [36]. Prolonged wakefulness (sleep deprivation) is associated with enhanced production of IL1β and TNFα [37–38]. Inhibition or inactivation of these cytokines with antibodies, receptor antagonists or soluble receptors reduces spontaneous NREMS as well as rebound sleep observed after sleep deprivation [39–42]. Furthermore, mice lacking both IL1β type 1 receptor and the TNFα type 1 receptor show reduced NREMS increase after sleep deprivation [43]. These findings suggest that increased production of pro-inflammatory cytokines in response to sleep loss are important metabolic signals for recovery sleep after sleep loss. Reduced cytokine production due to the reduced number of M1 macrophages in macrophage-depleted mice likely contributes to the attenuation of sleep rebound after sleep loss.

The activated BAT is an important source of sleep-promoting signals after sleep loss and in cold environment. Pharmacological stimulation of BAT induces sleep [44]. Sleep deprivation causes the activation of BAT [11]. Compensatory, rebound sleep responses are greatly reduced in transgenic mice with dysfunctional BAT thermogenesis [11]. Since M2 macrophages play a key role in stimulating BAT thermogenesis [9], it is possible that in addition to M1 cells, M2 macrophages may also have sleep-promoting actions by acting on the BAT. Sleep loss sets the molecular machinery of M2 cell activation in motion in BAT indicated by the increased expression of IL4 mRNA (Szentirmai and Kapás, unpublished). This finding indirectly supports the notion that sleep loss-induced sleep rebound is possibly M2 cell dependent. Also, intact BAT function is required for regulated heat production in the cold (reviewed in [3]). Exposure to lower ambient temperature is associated with the polarization of macrophages to the M2 state in both white and brown adipose tissues. M2 macrophages are necessary for BAT thermogenesis as indicated by the severe cold intolerance of mice deficient of M2 cells [9]. Taken together, these findings suggest that the lack of M2 macrophages are likely the cause of the augmented suppression of sleep and body temperature in cold environment in macrophage-deficient mice.

We also investigated the acute effects of CCL treatment on sleep and body temperature. CCL treatment had an immediate and robust effect on sleep and body temperature. Macrophages are major sources of pro-inflammatory cytokines and also various intracellular lysosomal enzymes [45]. Although plasma levels of cytokines were not measured after CCL treatment, it is safe to speculate that they were released from the disintegrating macrophages. It is likely that the acute effects of CCL on sleep and temperature are due to the release of these bioactive substances from the disintegrating M1 and M2 macrophages. The time-course and magnitude of fever, increased NREMS and suppressed REMS and EEG SWA after CCL injection are very similar to those observed after the administration of LPS and pro-inflammatory cytokines such as IL-1β and TNFα [46–48] and are consistent with an acute inflammatory response. Norepinephrine, released from BAT resident M2 macrophages, stimulates BAT activity via β3-adrenergic receptors. It is possible that in addition to the release of pro-inflammatory cytokines from M1 cells, norepinephrine release from disintegrating M2 macrophages also contributes to the acute sleep and febrile responses to CCL through activating BAT. Two days after systemic CCL treatment, remnants of macrophages are cleared [49] and serum concentrations of released molecules return to normal values [13]. Consistent with these findings, sleep, body temperature and motor activity returned to baseline by day 6 after CCL injections.

In conclusion, our finding that macrophage-depleted mice are unable to mount normal recovery sleep after sleep loss and have reduced sleep in cold environment strongly supports the idea that macrophages are important sources of peripheral signals required for normal sleep regulation. Further studies with site-specific depletion of macrophages, and isolated depletion of M1 and M2 macrophages are required to understand the relative contribution of various macrophage pools to sleep signaling.
